# Supplementary material for: Gap junctions amplify spatial variations in cell volume in proliferating tumor spheroids
Source: Nat Commun. 2020 Dec 1;11:6148. doi: 10.1038/s41467-020-19904-5 (PMC7708487; doi:10.1038/s41467-020-19904-5)
Supplement: Supplementary file 1 — Supplementary Information [file 41467_2020_19904_MOESM1_ESM.pdf]

Supplementary Note 1. Thermodynamically motivated derivation of permeability equations

Here we derive equations of solute and solvent flow between connected solutions, as per the work of Kedem *et al.* (1958)[1]. As highlighted by Kedem *et al.*, entropy production is the starting point of any thermodynamic description of non-equilibrium systems. Initially considering a 2-cell system in which solutions are separated by a membrane, entropy production d_iS/dt is given by

$$\frac{d_iS}{dt} = \frac{1}{T}\left(\mu_w^{i+1} - \mu_w^i\right)\frac{dN_w^i}{dt} + \frac{1}{T}\left(\mu_s^{i+1} - \mu_s^i\right)\frac{dN_s^i}{dt}\ , \qquad (S1)$$

where T is the absolute temperature, μ_w (μ_s) denotes the chemical potential of the solvent (solute), and dN/dt is associated number of moles passing into cell i per unit time. The dissipation (per unit area) may then be written as

$$\Phi = \frac{\mathrm{T}}{\mathrm{A}}\frac{d_iS}{dt} = \left(\mu_w^{i+1} - \mu_w^i\right)\dot{n}_w + \left(\mu_s^{i+1} - \mu_s^i\right)\dot{n}_s, \qquad (S2)$$

where $\dot{n}_w = (1/A)dN_w^i/dt$ and $\dot{n}_s = (1/A)dN_s^i/dt$. With an approximation that the chemical potentials for ideal solutions are appropriate, the chemical potential difference is given by

$$\mu^{i+1} - \mu^i = \bar{V}\Delta P + RT\Delta\ln(y)\,, \qquad (S3)$$

such that $\bar{V}$ is the partial molar volume, ΔP is the hydrostatic pressure difference between the cells, y is the molar fraction of the constituent, and R is the gas constant. In a dilute solution, where the volume fraction of the solute is relatively small (i.e. $c_s\bar{V}_s \ll 1$), Eqn S3 can be rewritten as

$$\mu_s^{i+1} - \mu_s^i = \bar{V}_s\Delta P + RT\Delta\ln(c_s) = \bar{V}_s\Delta P + RT\frac{\Delta c_s}{c_s^*}, \qquad (S4)$$

where c_s^* is the mean solute concentration across both cells and $\Delta c_s/c_s^* = \ln(c_s^{i+1}/c_s^i)$ if $\Delta c_s/c_s^i \ll 1$. Similarly, the chemical potential difference for the solvent is

$$\mu_w^{i+1} - \mu_w^i = \bar{V}_s\Delta P + RT\frac{\Delta c_s}{c_w}. \qquad (S5)$$

where to good approximation, $c_w = 1/\bar{V}_w$. We can thus modify our dissipation function such that

$$\Phi = (\dot{n}_w\bar{V}_w + \dot{n}_s\bar{V}_s)\Delta P + \left(\frac{\dot{n}_s}{c_s^*} - \frac{\dot{n}_w}{c_w}\right)\Delta\Pi, \qquad (S6)$$

whereby the osmotic pressure difference $\Delta\Pi = RT\Delta\mathrm{c_s}$ via van't Hoffs relation. Considering this function represents a special case of the general expression $\Phi = \sum_i J_iX_i$, where J_i denotes a flow and X_i is the generalized conjugated force, the forces $X_v = \Delta P$ and $X_D = \Delta\Pi$ identify the conjugate flows $J_v = \dot{n}_w\bar{V}_w + \dot{n}_s\bar{V}_s$ and $J_D = \dot{n}_s/c_s^* - \dot{n}_w/c_w$. Following the general theory of irreversible thermodynamics and Onsagar reciprocal relations[2], the flows may also be expressed as:

$$J_v = L_p\Delta P + L_{pD}\Delta\Pi \qquad (S7)$$

$$J_D = L_{Dp}\Delta P + L_D\Delta\Pi,$$

where the L's are phenomenological coefficients that govern the membrane permeability and $L_{Dp} = L_{pD}$. Kedem *et al*[1] further highlighted that these coefficients could be related through the Staverman reflection coefficient[3], o_r, leading to $L_{pD} = -o_rL_p$. This coefficient indicates the level of membrane selectivity, with $o_r = 1$ denoting an ideally selective (channels non-permeable to solutes) membrane

and $o_r = 0$ a fully non-selective membrane. We note the reflection coefficient is typically denoted by σ but is instead here represented by o_r to avoid confusion with the standard notation for stress. They proposed that the flow equations can thus be rearranged such that

$$J_v = L_p(\Delta P - o_r \Delta \Pi) \quad \text{and} \tag{S8}$$

$$\dot{n}_s = c_s^* L_p (1 - o_r)\Delta P + \left[\omega - c_s^* L_p (1 - o_r) o_r\right]\Delta \Pi, \tag{S9}$$

where ω is a coefficient that relates to solute permeability.

Supplementary Note 2. Gap junction mediated solvent and ion transport between cells

To approach the problem of multicellular volume regulation, we consider two cells held together via cadherin and catenin mediated complexes. As these complexes stabilize on the membrane, connexin structures also assemble and couple with identical units on the neighbouring cell to form gap junctions (GJs). These channels connect the cytoplasm of both cells, permitting passive transport of solvent, ions, and small molecules[4]. GJs typically remain open during their lifecycle, though may close in response to high Ca2+ concentrations or low pH which serves to protect the cell from dying neighbors[5]. With a diameter in the range of $1.5 - 2\,nm$, GJs may be approximated as fully non-selective to water molecules (diameter $\approx 0.275\,nm$) and ions (diameters $\approx 0.1 - 0.2\,nm$) such that $o_r = 0$ [6]. Eqns S8 and S9 then reduce to

$$J_{v,g,i} = -L_{p,g}(P_i - P_{i+1}) \quad \text{and} \tag{S10}$$

$$\dot{n}_{g,i} = -c_s^* L_{p,g}(P_i - P_{i+1}) - \omega_g(\Pi_i - \Pi_{i+1}). \tag{S11}$$

The change in cellular volume associated with fluid flow through GJs may then be written as

$$\frac{dV_{c,i}}{dt} = A_g J_{v,g,i} = -A_g L_{p,g}(P_i - P_{i+1}), \tag{S12}$$

where A_g is the surface area of the connected membrane. Similarly the rate of change in the total number of ions in a cell is then given by

$$\frac{dN_i}{dt} = A_g \dot{n}_{g,i} = -A_g\left(c_s^* L_{p,g}(P_i - P_{i+1}) + \omega_g(\Pi_i - \Pi_{i+1})\right), \tag{S13}$$

where the first bracketed term accounts for advective ion flow and the second term describes diffusive transport.

Supplementary Note 3. Fluid and ion exchange with the extracellular environment

In addition to diffusion across gap junctions, water molecules can move through the semi-permeable cell membrane from the external media, enhanced by the presence of aquaporins[7]. As these pathways do not permit the diffusion of ions we can consider them to be fully selective (i.e. $o_r = 1$). We assume that the ion concentration in the external media is uniform, such that the external osmotic pressure at any point is given by Π^{ext}. From Eqn S8 the solvent volume flux through the membrane is then given by $J_{v,m,i} = -L_{p,m}(\Delta P_i - \Delta \Pi_i)$, where $\Delta P_i = P_i - P^{ext}$ and $\Delta \Pi_i = \Pi_i - \Pi^{ext}$. $L_{p,m}$ is the permeability coefficient associated with solvent flow through the membrane. Evidently, this flux depends on the difference in osmotic and hydrostatic pressure

between the cell and the extracellular environment. We can extend Eqn S12 to consider this additional water flux such that $dV_{c,i}/dt = A_g J_{v,g,i} + A_i J_{v,m,i}$. Note that with our assumption of uniform external hydrostatic and osmotic pressures (e.g. $P_i^{ext} = P_{i+1}^{ext} = P^{ext}$) we can also state the gap junction flux as a function of pressure differences, such that $J_{v,g,i} = -L_{p,g}(\Delta P_i - \Delta P_{i-1})$. Assuming the cells can be approximated to retain a spherical shape with radius $r_{c,i}$, we achieve the following expanded form for cellular volume change:

$$\frac{dV_{c,i}}{dt} = -A_g L_{p,g}(\Delta P_i - \Delta P_{i+1}) -A_i L_{p,m}(\Delta P_i - \Delta \Pi_i), \qquad (S14)$$

where $A_i = 4\pi r_{c,i}^2$ is the cell surface area. The cytosolic ion concentration also depends on exchange with the extracellular environment through selective ion channels. As these channels do not facilitate solvent flow the associated fluxes assume the general form $\dot{n}_i = \omega \Delta \Pi_i$. Mechanosensitive (MS) channels are proteins in the cell membrane that open under a tensile membrane stress[8] to allow flow of ions from regions where the concentration is high to regions where it is low. In response to hypotonic shock they release ions from the cell to mitigate an influx of water. The probability of channel opening has been reported to follow a Boltzmann function[9], and consistent with previous work[10], we adopt a piecewise linear expression (Fig 1, yellow curve) to describe the ion flux associated with MS channel permeability $\dot{n}_{ms,i} = -\omega_{ms}(\sigma_i)\Delta \Pi_i$, such that

$$\omega_{ms}(\sigma_i) = \begin{cases} 0 & if\ \sigma_i \leq \sigma_c \\ \beta(\sigma_i - \sigma_c) & if\ \sigma_c < \sigma_i < \sigma_s \\ \beta(\sigma_s - \sigma_c) & if\ \sigma_i \geq \sigma_s \end{cases}, \qquad (S15)$$

where σ_i is the computed cortical stress (detailed in Section S4), σ_c is the threshold stress, below which $\dot{n}_{ms,i} = 0$, σ_s is the saturating stress, above which the channels are fully open, and β is a rate constant. In addition to these force sensitive channels, there are a number of leak channels (which are always operative) on the membrane[11] for which we consider an further transmembrane ion flux $\dot{n}_{l,i} = -\omega_l \Delta \Pi_i$, where ω_l is the associated permeability coefficient. While the channels described thus far permit passive ion diffusion, there are additional membrane proteins present that actively transport ions against the concentration gradient. These ion pumps require an energy input, such as from ATP hydrolysis, to overcome the energetic barrier associated with moving ions against the concentration gradient. Following Jiang and Sun (2013)[10], the free energy change associated with pumping action can be expressed as $\Delta G = RT \log(c_i/c_{ext}) - \Delta G_a$, where ΔG_a is an energy input is associated with hydrolysis of ATP. The ion flux associated with active pumping can then be written as $\dot{n}_{p,i} = \gamma' \Delta G$, where γ' is a permeation constant. Maintaining our dilute assumption, ΔG can be linearized as $\Delta G = RT\,(\Pi_i - \Pi^{ext})/\Pi^{ext} - \Delta G_a$. We can therefore identify a critical osmotic pressure difference $\Delta \Pi_c$, determined when $\Delta G = 0$, such that $\Delta \Pi_c = \Pi^{ext} \Delta G_a / RT$ (noting that when $\Delta G > 0$ active pumping is no longer energetically favorable and the pumping direction will reverse[12]). Thus the ion flux generated by active pumping by ion transporters can be expressed as $\dot{n}_{p,i} = \gamma(\Delta \Pi_c - \Delta \Pi_i)$, where γ is a rate constant. Taking these pumps and channels into consideration, we can extend Eqn S13 for a more detailed description of the number of ions within the cell whereby $dN_i/dt = A_g \dot{n}_{g,i} + A_i(\dot{n}_{ms,i} + \dot{n}_{l,i} + \dot{n}_{p,i})$ such that

$$\frac{dN_i}{dt} = -A_g\left(c_s^* L_{p,g}(\Delta P_i - \Delta P_{i+1}) + \omega_g(\Delta \Pi_i - \Delta \Pi_{i+1})\right) -A_i\big((\omega_{ms}(\sigma_i) + \omega_l + \gamma)\Delta \Pi_i - \gamma \Delta \Pi_c\big). \qquad (S16)$$

Clearly the mean ion concentration between two connected cells, c_s^*, can be expressed in terms of osmotic pressure as $c_s^* = (c_i + c_{i+1})/2 = (\Pi_i + \Pi_{i+1})/(2\,RT)$. Based on our established terminology whereby $\Delta\Pi_i = \Pi_i - \Pi^{ext}$, this can be rephrased such that $c_s^* = (\Delta\Pi_i + \Delta\Pi_{i+1} + 2\Pi^{ext})/(2\,RT)$. Assuming that $\Delta\Pi_i/\Pi^{ext} \ll 1$, for the calculation of solute flow we can therefore approximate $c_s^* \approx \Pi^{ext}/(RT)$ and Eqn S16 can be rephrased as

$$\frac{dN_i}{dt} = -A_g\left(\frac{\Pi^{ext}}{RT}L_{p,g}(\Delta P_i - \Delta P_{i+1}) + \omega_g(\Delta\Pi_i - \Delta\Pi_{i+1})\right) - A_i\big((\omega_{ms}(\sigma_i) + \omega_l + \gamma)\Delta\Pi_i - \gamma\Delta\Pi_c\big). \qquad (S17)$$

Material parameters for all simulations are summarized in Supplementary Table 1.

Supplementary Note 4. Motivations for mechanical model of cell cortex

As water enters a cell, driven by hydrostatic and osmotic pressure gradients, the increase in fluid volume stretches the cell membrane. The mechanical tension in the membrane is complex, controlled by membrane-cytoskeleton adhesion, cortical stiffness, and active myosin contractility[13,14]. We treat the membrane and cortex as a single mechanical structure[10], neglecting the possibility of cortical detachment and blebbing. The constitutive law of the cortical structure can be written as $\sigma_i = \sigma_{p,i} + \sigma_{a,i}$, where $\sigma_{a,i}$ is the active stress associated with myosin contractility and $\sigma_{p,i}$ is the passive stress predominantly associated with deformation of the actin network (as the actin cortex is much stiffer than the plasma membrane[13,15]). As per previous cortex models[10,16,17], we begin with the consideration of a general viscoelastic model such that the passive stress may be expressed by $\sigma_{p,i} = K(A_i/A_0 - 1)/2 + \eta(1/A_i)(dA_i/dt)$, where K is the effective cortical stiffness, η is the effective viscosity of the cell cortex, and A_0 is a reference surface area. The apparent cortex viscosity has been reported to lie in the range of $10^2 - 10^3\ Pa.s$ [18,19], and under loading the cell radius has been reported to change by ~10% in several minutes[20]. As such, the viscous stress $\eta(2/r)(dr/dt) \approx 0.1 - 1\,Pa$ is much smaller than the elastic terms in the passive stress expression. Therefore, we consider the contribution of the viscous term to be negligible in this analysis with a view that K denotes the long-term cortical stiffness, and the total stress reduces to $\sigma_i = (K/2)(r_{c,i}^2/r_o^2 - 1) + \sigma_{a,i}$ for a spherical cell of radius $r_{c,i}$. An investigation more directed to the specific influence of cortical organization and myosin contractility could readily extend this expression to describe long-term remodeling in response to signaling and stress in accordance with our previous work[21,22].

In addition to internal fluid pressure, the membrane also experiences loading from a spatially uniform external fluid pressure P^{ext}. Mechanical force balance for a spherical cell with radius $r_{c,i}$ dictates that the cortical stress can be related to the pressure difference across the membrane $\Delta P_i = P_i - P^{ext}$. Therefore, the cortical stress may also be written as $\sigma_i = \Delta P_i r_{c,i}/2h_i$, where h_i is the cortical thickness. Further, within a multicellular organoid, proliferation of cells generates compressive solid stresses $\sigma_{g,i}$ that act on neighboring cells[23]. Deformation of fibrous matrix surrounding the cell cluster compounds the stress, as stretched fibers squeeze on the cluster[24]. Thus, we obtain the following expanded expression for the membrane/cortical stress:

$$\sigma_i = \frac{K}{2}\left(\frac{r_{c,i}^2}{r_0^2} - 1\right) + \sigma_{a,i} = \frac{(\Delta P_i - \sigma_{g,i})r_{c,i}}{2h_i}, \qquad (S18)$$

where r_0 is the reference cell radius. Further, we note that the passive stress expression may be linearized to reduce solution complexity by assuming that the cortical deformation is small such that $\sigma_{p,i} = (K/2)(r_{c,i}^2/r_o^2 - 1) = (K/2)((r_0^2 + 2\Delta r r_0 + \Delta r^2)/r_o^2) \approx K\big(r_{c,i}/r_0 - 1\big)$.

Supplementary Note 5. Pressure and fluxes from 2-cell analyses

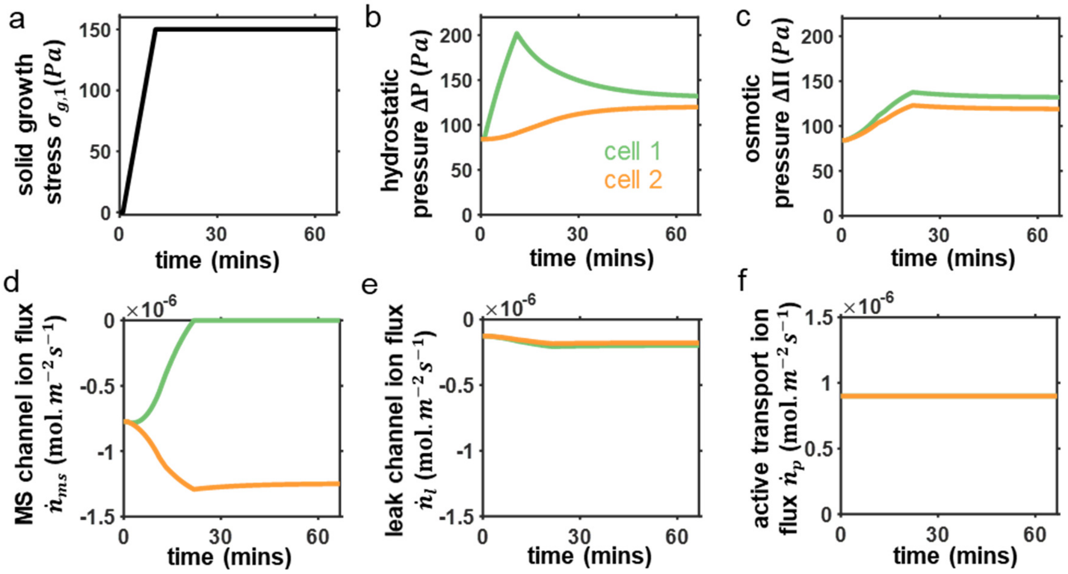

Supplementary Fig 1: ***Additional figures for two-cell analysis under control conditions*****. a) Form of solid growth stress applied to cell 1; Difference between internal and external b) hydrostatic pressure ΔP and c) osmotic pressure $\Delta\Pi$; Ion fluxes across the cell membrane: d) MS channels $\dot{n}_{ms}$; e) leak channels $\dot{n}_l$; f) active transport $\dot{n}_p$.**

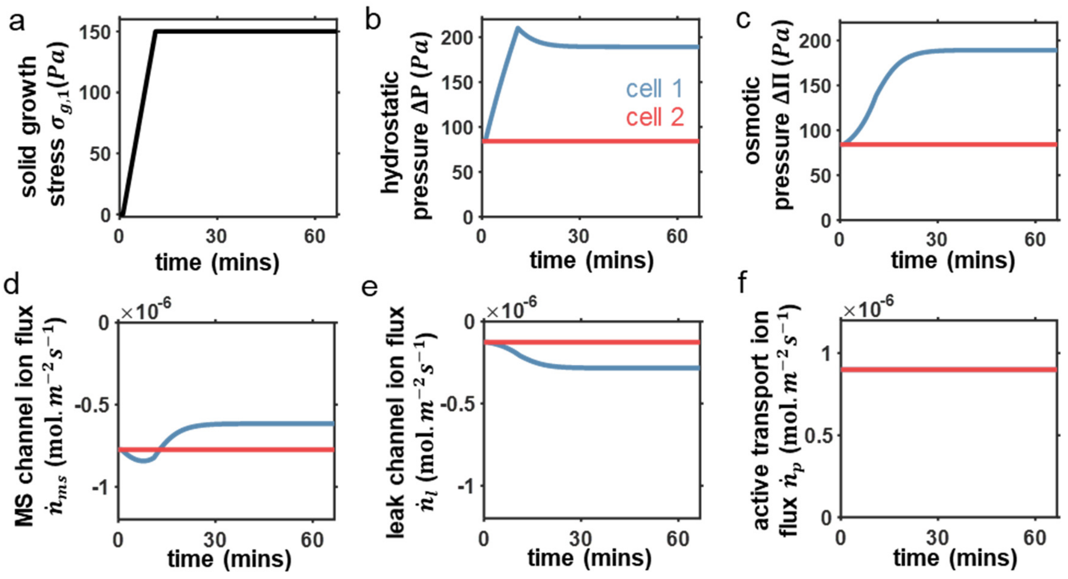

Supplementary Fig 2: ***Additional figures for two-cell analysis during inhibition of ion flux across gap junctions*****. a) Form of solid growth stress applied to cell 1; Difference between internal and external b) hydrostatic pressure ΔP and c) osmotic pressure $\Delta\Pi$; Ion fluxes across the cell membrane: d) MS channels $\dot{n}_{ms}$; e) leak channels $\dot{n}_l$; f) active transport $\dot{n}_p$.**

Supplementary Note 6. Material parameters and motivation

Parameter	Description	Value
ω_g	gap junction ion permeability factor ($mol.m^{-2}s^{-1}Pa^{-1}$)	$5\,X\,10^{-7}$
$L_{p,g}$	gap junction water permeability factor ($m.s^{-1}Pa^{-1}$)	$1\,X\,10^{-11}$
r_0	reference cell radius (μm)	7.1
A_g	area of membrane adhered between two cells (μm^2)	63.3
K	effective stiffness of cortical layer (kPa)	6
σ_a	active cortical stress (Pa)	100
h	thickness of cortical layer (μm)	0.6
β	MS channel ion permeability factor ($mol.m^{-2}s^{-1}Pa^{-2}$)	$2\,X\,10^{-11}$
σ_c	threshold stress of MS channel (Pa)	75
σ_s	saturating stress of MS channel (Pa)	600
ω_l	leak channel ion permeability factor ($mol.m^{-2}s^{-1}Pa^{-1}$)	$1.5\,X\,10^{-9}$
γ	rate constant of ion flux via active transporters ($mol.m^{-2}s^{-1}Pa^{-1}$)	$2.25\,X\,10^{-17}$
$\Delta\Pi_c$	critical osmotic pressure difference of ion pump (GPa)	40
Π^{ext}	external osmotic pressure (MPa)	0.67
$L_{p,m}$	cell membrane water permeability factor ($m.s^{-1}Pa^{-1}$)	$7\,X\,10^{-12}$
r_{max}	spheroid radius (μm)	33.4

Supplementary Table 1: ***Parameters for chemo-osmotic model.***

The density of GJs on the adhered membrane of epithelial-like cells has been estimated[25] to lie in the range of $\rho_g \approx 2\,\text{X}\,10^{14}\,m^{-2}$. Assuming Poiseuille flow through GJs of radius $r_g \approx 1\,nm$ and length $l_g \approx 15\,nm$ [26], following Mathias *et al* (2008)[25] and Gao *et al.* (2011)[6], the solute permeability factor can be estimated as $L_{p,g} = \rho_g \pi r_g/(8\eta l_g)$, where η is the viscosity of water. Within channels of radius $1\,nm$ and at body temperature ($T = 310\,K$), the viscosity of water can be approximated as $0.5\,mPa.s$ [27], leading to a coefficient $L_{p,g} \approx 10^{-11}\,m.s^{-1}.Pa^{-1}$. Further, considering the molar volume of water $\bar{V}_w = 18\,mL/mol$, we can determine the solvent GJ permeability coefficient in terms of the number of moles whereby $\omega_{g,w} = L_{p,g}/\bar{V}_w = 5.82\,\text{X}\,10^{-7}\,mol.m^{-2}s^{-1}Pa^{-1}$. Assuming similar diffusive behavior for the solutes (ions), $\omega_{g,w}$ suggests that ion coefficient ω_g should lie in the range $10^{-7} - 10^{-6}\,mol.m^{-2}s^{-1}Pa^{-1}$. We determined that a value of $\omega_g = 5\,\text{X}\,10^{-7}\,mol.m^{-2}s^{-1}Pa^{-1}$ provides good agreement between our simulated and experimentally measured volumes, assuming a cell-cell adhered membrane surface area A_g on the order of 10% of the reference cell surface. Across a number of cell types the membrane solvent permeability rate P_s has been reported to lie in the range of $10^{-4} - 10^{-5}\,m/s$ [28]. Jiang and Sun (2013)[10] highlighted that this can be related to the membrane water permeability factor such that $L_{p,m} = P_s\bar{V}_w/RT$, from which we ascertain an upper value of $L_{p,m} = 7\,X\,10^{-12}$ used here. The spheroid radius r_{max} is directly measured from the day 5 experimental images (Fig 3). In epithelial cells, the thickness h of the actin cortex has been reported to vary from $0.1 \sim 0.6\,\mu m$[29] for which we assume the upper value. Experimentally reported values for cell stiffness are highly variable (0.1 – 100 kPa [30]), likely associated with

measurement timescales and cell remodeling. We assume a value $K = 6\,kPa$ and $\sigma_a = 100\,Pa$ in line with Jiang and Sun (2013), with treatment of dissipative effects discussed in Section S4. The reference cell radius r_0 was approximated such that the prediced cell volumes at an early stage of organoid growth provide good agreement with our experiments (Supplementary Fig 10e). The external osmotic pressure Π^{ext} can be computed from physiological ion concentrations ($c_{Na^+,e} = 145mM$, $c_{K^+,e} = 5mM$ and $c_{Cl^+,e} = 110mM$ [31]) with $\Pi^{ext} = RT\left(c_{Na^+,e} + c_{K^+,e} + c_{Cl^-,e}\right) = 0.67\,MPa$. As the free energy from ATP is $\Delta G_a \approx 30\,kJ/mol$, the critical osmotic pressure for active pumping is then $\Delta\Pi_c = \Pi^{ext}\Delta G_a/RT = 40\,GPa$. The flux associated with active ion pumps has been measured to lie between 10^{-7} and 10^{-6} $mol.m^2s^{-1}$ [32,33]. As per Jiang and Sun (2013), after dividing by $\Delta\Pi_c$ the pump coefficient should be confined to the range $\gamma \approx 2.5\,X\,10^{-18} \sim 2.5\,X\,10^{-17}$ $mol.m^{-2}s^{-1}Pa^{-1}$, for which we choose a value of $2.25\,X\,10^{-17}$ $mol.m^{-2}s^{-1}Pa^{-1}$. The ion fluxes across mechanosensitive and leak channels have an amplitude on the order of active ion pumps (i.e. $10^{-7} \sim 10^{-6}$ $mol.m^2s^{-1}$)[32,33]; To maintain such continuity Jiang and Sun (2013) suggested a value $\beta = 2\,X\,10^{-11}$ $mol.m^{-2}s^{-1}Pa^{-2}$, which we retain for our analyses, while the threshold and saturation stress are reduced to provide better agreement with our experiments (Fig 3). In keeping with this motivation, the leak channel permeability factor ω_l is assumed to equal the threshold permeability of the MS channels (i.e. $\beta\sigma_c$). The influence of key model parameters on cell volume in two connected cells (in response to solid growth stress $\sigma_{g,1} =$

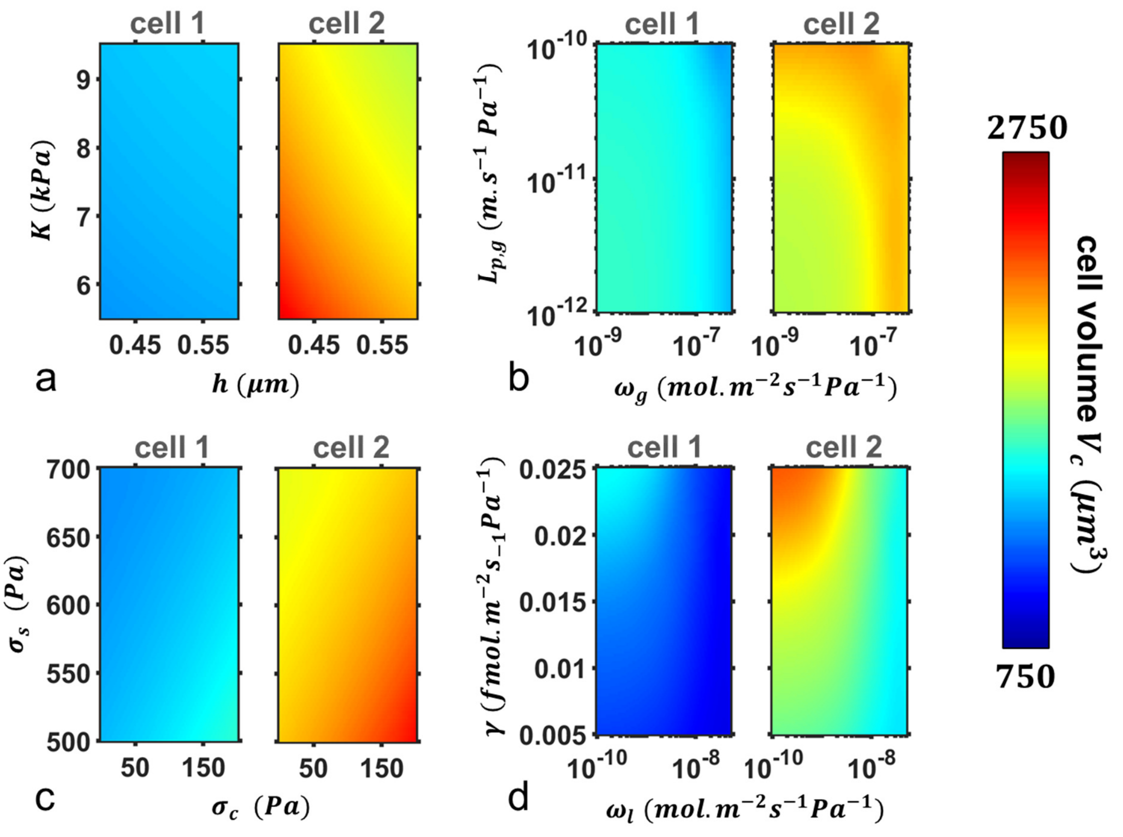

Supplementary Fig 3: ***Sensitivity of model predictions to key parameters*****. Cell volume changes in response to an applied solid growth stress $\sigma_{g,1} = 150\,Pa$ on cell 1 associated with variance in a) cortical stiffness K and thickness h, b) gap junction permeability to fluid $L_{p,g}$ and ions ω_g, c) mechanosensitive threshold stresses σ_c and σ_s, and d) pump coefficient γ and leak channel permeability ω_l.**

150 Pa on cell 1) is shown in Supplementary Fig 3. Briefly, increasing the effective cortical compliance (via a reduction in K or h) will lead to an increase in cell volume (Supplementary Fig 3a). Under loading, increasing the permeability of GJs to ions (via ω_g) will cause increased swelling of the connected neighbor (Supplementary Fig 3b); this behavior also emerges from increasing the permeability to solvent (via $L_{p,g}$) due to an increase in advective flow. Increasing the threshold stress σ_c for the opening of MS channels causes cell volume to increase (due to higher ion retainment), while increasing the stress at which MS channel opening saturates σ_s will reduce cell volume (Supplementary Fig 3c); this is akin to increasing channel permeability. Finally, increasing the rate of active pumping via γ will increase cell volume, while increasing the permeability of leak channels via ω_l will reduce cell volume (Supplementary Fig 3d).

Supplementary Note 7. Influence of cell elongation

In our main analyses we assume that the cells retain an approximately spherical shape. However, in proliferating clusters cells may attain high aspect ratios and more irregular configurations. In this section we propose a model extensio n to analyze highly elongated shapes for which a cylindrical geometry is a reasonable representation. In a thin-walled cylindrical vessel, the circumferential and longitudinal stress can be related to the pressure difference across the wall by $\sigma_{\theta,i} = \Delta P_i r_i / h_i$ and $\sigma_{l,i} = \Delta P_i r_i / 2h_i$, respectively. From classic Hookean relations, the change in radius and cylinder length may then be expressed as

$$\Delta r_i = \frac{r_0 \Delta P_i r_i}{K h_i}\left(1 - \frac{\nu}{2}\right), \qquad and$$

$$\Delta l_i = \frac{l_0 \Delta P_i r_i}{K h_i}\left(\frac{1}{2} - \nu\right) \qquad (S19)$$

respectively, where l_0 is the initial cylindrical length and ν is the material Poisson's ratio. Initially considering an arbitrar y cylinder comprised of an incompressible material ($\nu = 0.5$) and of fixed reference volume V_0, we find that with increasing reference length to radius ratio l_0/r_0 the cylinder becomes increasingly resistant to volume change under an equivalent load (Supplementary Fig 4a).

We next extend our mechano-osmotic model to simulate the behavior of elongated cylindrical cells in response to an applied growth stress. Combining Eqns S18 and S19 the change in cellular radius can be

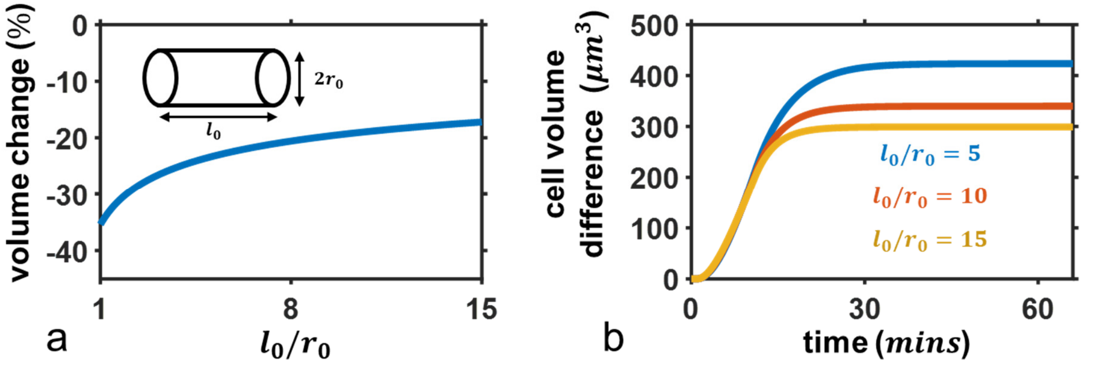

Supplementary Fig 4: ***Influence of cell elongation.*** **a) Influence of cylindrical length to radius ratio l_0/r_0 on volume change from a fixed reference volume $V_0 = 1500\ \mu m^3$ under constant pressure with $\Delta P/K = 0.025$; b) Difference in volume $V_2 - V_1$ of connected cylindrical cells under an applied solid growth stress $\sigma_{g,1} = 150\ Pa$.**

expressed as $\Delta r_i = (r_0/K)(3\Delta P_i r_i/(4h_i) \; - \sigma_{a,i}/2)$. Assuming for illustrative purposes that the cortex is incompressible, the length change then reduces to $\Delta l_i = h_0\sigma_{a,i}/2K$ due to the Poisson effect. For the flux relations, the cellular surface area $A_i = 2\pi l r_i + 2\pi r_i^2$ and we assume that the opening of mechanosensitive channels (via Eqn S15) depends predominantly on the circumferential stress as $\sigma_{\theta,i} = 2\sigma_{l,i}$. All other model elements and parameters remain as defined for the standard mechano-osmotic model. Under an applied solid growth str ess σ_g, a volume difference between the loaded and unloaded cell emerges as per our analysis of spherical cells. However, in keeping with the mechanisms highlighte d for an arbitrary cylinder, with an increasing reference length to radius ratio l_0/r_0 the volume difference is predicted to reduce (Supplementary Fig 4b). This indicates that, as cells elongate, they become less sensitive to loading. Beyond, our model may be extended for the consideration of more erratic and complex cell shapes, but such studies will require discrete geometry definitions and finite element analysis to describe local variations in wall stretch and solute flux.

Supplementary Note 8. Mechano-electro-osmotic solute flow in single and connected cells

Consideration of electrical potentials: Up until this point we have confined our consideration of cellular volume regulation to a dependence on osmotic and hydrostatic pressures, and neglected the role of electrical potentials. In this section, we proceed to define a framework for coupled mechano-electro-osmotic (MEO) interactions and demonstrate that the mechanisms proposed for multi-cell volume regulation are largely unchanged by such an extension. We begin by introducing the classic pump-leak model[34,35] to describe the fluxes of dominant ions associated with cellular volume control (Na^+, K^+, and Cl^-), whereby

$$\frac{dc_{Na^+,i}}{dt} = -\frac{A_i g_{Na}}{FV_i}\left(\phi_i - \frac{RT}{F}\ln\left(\frac{c_{Na^+,e}}{c_{Na^+,i}}\right)\right) - \frac{3pA_i}{FV_i} \;, \qquad (S20)$$

$$\frac{dc_{K^+,i}}{dt} = -\frac{A_i g_K}{FV_i}\left(\phi_i - \frac{RT}{F}\ln\left(\frac{c_{K^+,e}}{c_{K^+,i}}\right)\right) + \frac{2pA_i}{FV_i} \;, \qquad (S21)$$

$$\frac{dc_{Cl^-,i}}{dt} = \frac{A_i g_{Cl}}{FV_i}\left(\phi_i + \frac{RT}{F}\ln\left(\frac{c_{Cl^-,e}}{c_{Cl^-,i}}\right)\right) . \qquad (S22)$$

Here, solute concentrations $c_{j,i} = N_{j,i}/V_i$, ϕ_i is the membrane potential, A_i is the cell surface area, F is the Faraday constant, and g_j are the ion channel conductances for a given species. The log-based term is a form of the Nernst equation, determining the electrical potential of the species as a function of internal and external (e) concentrations. In this formulation, p is the strength of the pump current with associated values representing the 3:2 stoichiometry of the $Na - K$ ATPase. While earlier we assumed that pumping actively transports a single ion species across the membrane, the biological mechanism of $Na - K$ pumping is more complex. From Glitsch and Tappe (1995)[12], the free energy during the pumping action is $\Delta G = \Delta G_a + 3\left(-F\phi_i + RT\ln\left(\frac{c_{Na^+,e}}{c_{Na^+,i}}\right)\right) + 2\left(F\phi_i + RT\ln\left(\frac{c_{K^+,e}}{c_{K^+,i}}\right)\right)$, where ΔG_a is an energy input associated with hydrolysis of ATP. Provided this reaction is energetically favorable $(\Delta G < 0)$, the pump transfers three Na^+ ions against their concentration gradient to the cytosol, and two $K^+$from the external media into the cytosol. However, the net result of this complex pathway, which downstream is predicted to cause an overall increase in cytosolic osmotic pressure and cell volume (Supplementary Fig 5g-h), is similar to the behavior captured by only considering an active influx in Eqn 6.

The balance laws must be additionally supplemented by the electroneutrality condition

$$c_{Na^+,i} + c_{K^+,i} - c_{Cl^-,i} + z_x X/V_i = 0, \quad (S23)$$

where X is the total amount of impermeable molecules in the cell[35] which have a mean valence z_x. Initially considering a single suspended cell, from Eqn S8 the volume may thus be determined from $dV_i/dt = -L_{p,m}(\Delta P_i - \Delta\Pi_i)$, where the osmotic pressure difference $\Delta\Pi_i = RT(c_{Na^+,i} + c_{K^+,i} + c_{Cl^-,i} + X/V_i - (c_{Na^+,e} + c_{K^+,e} + c_{Cl^-,e}))$ and recall that the hydrostatic pressure difference $\Delta\mathrm{P}_i = 2\sigma_i h/r_i + \sigma_{g,i}$.

Predictions for single-cell behavior: Previously we have discussed mechanosensitive (MS) channels, proteins in the cell membrane that open under a tensile membrane stress to allow solute flow as driven by their electro-osmotic potential. One of the most widely appreciated MS pathways relates to Piezo1, which opens under tension to allow a calcium influx, thereby activating Ca^{2+}-gated K^+ channels to relieve osmotic pressure in swollen cells[36]. This behavior may be described by adapting our MS channel model (Eqn S15) to incorporate a stress-dependence in the conductivity of K^+ channels, such that

$$g_K(\sigma_i) = \begin{cases} g_{K0} & if\ \sigma_i \leq \sigma_c \\ g_{K0} + \beta_K(\sigma_i - \sigma_c) & if\ \sigma_c < \sigma_i < \sigma_s\,, \\ g_{K0} + \beta_K(\sigma_s - \sigma_c) & if\ \sigma_i \geq \sigma_s \end{cases} \quad (S24)$$

where g_{K0} is the conductivity of leak (always permeable) channels, and β_K, σ_c, and σ_s are MS constants. Finally, Mori (2011)[35] demonstrated that the membrane potential may be approximated by

$$\phi_i = \frac{FV_i}{C_m A_i}\left(c_{Na^+,i} + c_{K^+,i} - c_{Cl^-,i} + z_x \frac{X}{V_i}\right), \quad (S25)$$

where C_m is the membrane capacitance per unit area. This system of equations can be solved to predict the time-dependent evolution of cell volume, membrane potential, and solute concentrations using in-built ode solvers in Matlab (*ode23s*). We assume the external ion concentrations remain at physiological levels, such that $c_{Na^+,e} = 145mM$, $c_{K^+,e} = 5mM$ and $c_{Cl^+,e} = 110mM$ [31] and that the conductivity coefficients are confined to previously suggested ranges[37], with $C_m = 0.01\,F/m^2$, $g_{Na} = 0.1\,S/m^2$, $g_{Cl} = 2\,S/m^2$, and $g_K^{max} = 1\,S/m^2$ such that $g_{K0} + \beta_K(\sigma_s - \sigma_c) = g_K^{max}$ and $g_{K0} = 0.1\,S/m^3$. The pump current $p = 5\,mA/m^2$, number of impermeable solutes $X = 2\,X\,10^{-13}\,mol$, and mean valence $z_x = -1.5$ are estimated to provide a reasonable prediction of cytosolic ion concentrations (Supplementary Fig 5)[31]. The remining model parameters are fixed at those previously outlined (Supplementary Table 1). Following the application of stress σ_g, the cell is predicted to reduce in volume (Supplementary Fig 5a), with an associated steady state increase in membrane potential due to an increase in K^+ ions (Supplementary Fig 5c). The MEO equations may also be rearranged at steady state by setting the time derivatives to zero (i.e. $d/dt = 0$), reducing to the following:

$$Na_e e^{-\left(\frac{F\left(\phi+\frac{3p}{g_{Na}}\right)}{RT}\right)} + K_e e^{-\left(\frac{F\left(\phi-\frac{2p}{g_K}\right)}{RT}\right)} - Cl_e e^{\left(\frac{F\phi}{RT}\right)} + z_x \frac{X}{V_i} = 0, \quad (S26)$$

$$RT\left(Na_e e^{-\left(\frac{F\left(\phi+\frac{3p}{g_{Na}}\right)}{RT}\right)} + K_e e^{-\left(\frac{F\left(\phi-\frac{2p}{g_K}\right)}{RT}\right)} + Cl_e e^{\left(\frac{F\phi}{RT}\right)} + \frac{X}{V_i} - (Na_e + K_e + Cl_e) \right) - \Delta P_i = 0, \quad (S27)$$

where the first expression stems from the electroneutrality condition and the second from steady state hydrostatic and osmotic pressure balance. We solve this system using the Matlab in-built function

$fsolve$ (Supplementary Fig 5d-f) in conjunction with the mechanical constraints. This approach no longer requires the approximation for the membrane potential (Eqn S25), and shows good agreement with the transient solution (blue markers in Supplementary Fig 5d-f). It is interesting to note that with consideration of electrolyte flow, the magnitude of applied stress required to induce a comparable reduction in cell volume is significantly higher than in the case non-electrolyte flow. In non-electrolyte flow, an applied stress increases cytosolic hydrostatic pressure, causing a solvent efflux. In response to a reduced fluid content the cytosolic osmotic pressure increases and thus there is also an increased solute flow to the external media. In turn, additional fluid is lost due to a depleted cytosolic ion concentration. However, with inclusion of the role of fixed charges and electroneutrality, osmotic gradients alone are not sufficient to cause an equivalent solute flow, as the electrical potential within the cytosol resists ion loss. As such the osmotic pressure remains high under single cell loading, permitting the cell to retain fluid and resist shrinkage.

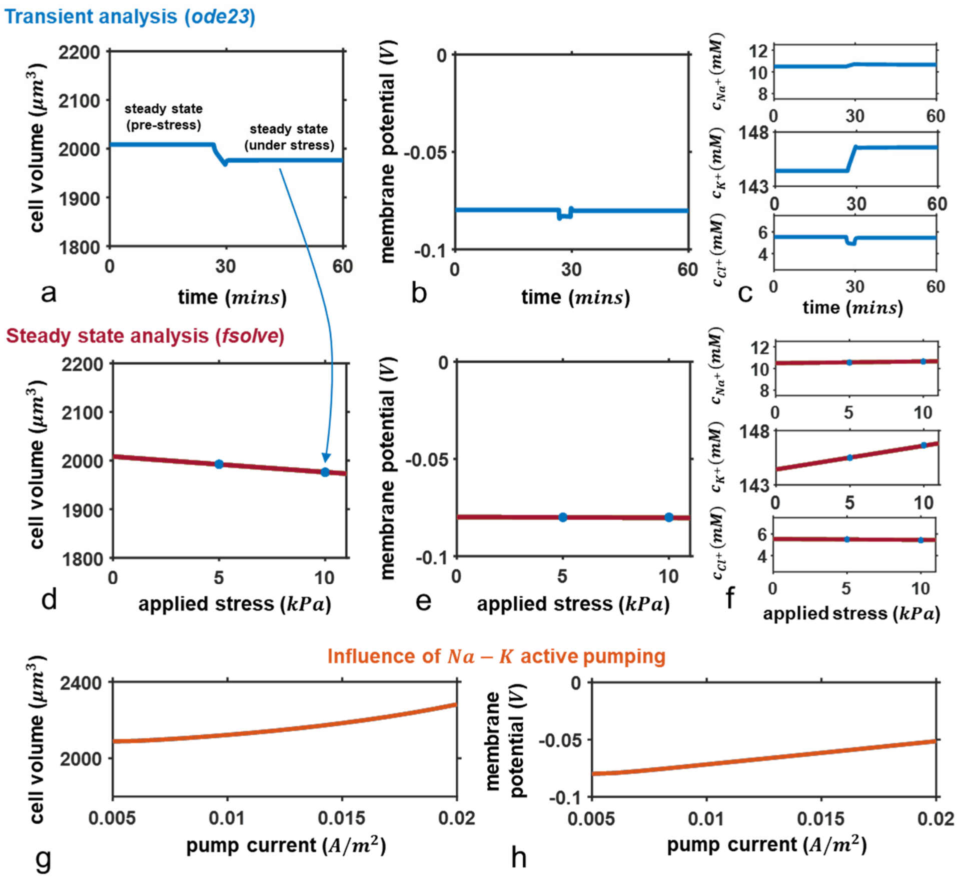

Supplementary Fig 5: ***Influence of mechano-electro-osmotic flow in single cells*. (a-c) Single cell behavior in response to applied loading with consideration of electro-osmotic flow using Matlab ode23s to compute the time-series solution: predictions for a) cell volume, b) membrane potential, and c) concentrations of Na^+, K^+, and Cl^+ over time. (d-f) Steady state single cell behavior in response to a series of applied loading with consideration of electro-osmotic flow using Matlab *fsolve:* predictions for d) cell volume, e) membrane potential, and f) concentrations of Na^+, K^+, and Cl^+. Markers show corresponding predictions at steady state from the transient analysis. (g-h) Influence of pump current p on g) cell volume and h) membrane potential.**

Analysis of connected cells: We next proceed to extend the MEO model for the analysis of connected cells and solvent/solute flow through gap junctions (GJs). As described in Section S2, GJs are not selective for individual ion species or solvent. Combining Eqns S11 and S20, transport of a given (positively charged) ion species j through GJs (from cell $i+1$ to cell i) may then be expressed by

$$\frac{dc_{g,j}}{dt} = -\frac{A_g g_g}{FV_i}\left((\phi_i - \phi_{i+1}) - \frac{RT}{F}\ln\left(\frac{c_{j,i+1}}{c_{j,i}}\right)\right) - \frac{A_g L_{p,g} c_j^*}{V_i}(\Delta P_i - \Delta P_{i+1}), \qquad (S28)$$

where the term on the left describes diffusive flow driven by electro-osmotic gradients and the term on the right describes advective flow as driven by hydrostatic pressure gradients (as described in Section S1). Here g_g is a coefficient associated with GJ conductivity, assumed for demonstrative purposes to equal g_K^{max}, and c_j^* is the mean cytosolic concentration of ion species j across the connected cells of interest. Eqns S20-S21 can then be extended to describe the change in ion concentrations within a given cell i as dependent on exchange across both the cell membrane and GJs, whereby:

$$\frac{dc_{Na^+,i}}{dt} = -\frac{A_i g_{Na}}{FV_i}\left(\phi_i - \frac{RT}{F}\ln\left(\frac{c_{Na^+,e}}{c_{Na^+,i}}\right)\right) - \frac{3pA_i}{FV_i}$$
$$-\frac{A_g g_g}{FV_i}\left((\phi_i - \phi_{i+1}) - \frac{RT}{F}\ln\left(\frac{c_{Na^+,i+1}}{c_{Na^+,i}}\right)\right) - \frac{A_g L_{p,g} c_{Na^+}^*}{V_i}(\Delta P_i - \Delta P_{i+1}), \qquad (S29)$$

$$\frac{dc_{K^+,i}}{dt} = -\frac{A_i g_K}{FV_i}\left(\phi_i - \frac{RT}{F}\ln\left(\frac{c_{K^+,e}}{c_{K^+,i}}\right)\right) + \frac{2pA_i}{FV_i}$$
$$-\frac{A_g g_g}{FV_i}\left((\phi_i - \phi_{i+1}) - \frac{RT}{F}\ln\left(\frac{c_{K^+,i+1}}{c_{K^+,i}}\right)\right) - \frac{A_g L_{p,g} c_{K^+}^*}{V_i}(\Delta P_i - \Delta P_{i+1}), \qquad (S30)$$

$$\frac{dc_{Cl^-,i}}{dt} = \frac{A_i g_{Cl}}{FV_i}\left(\phi_i + \frac{RT}{F}\ln\left(\frac{c_{Cl^-,e}}{c_{Cl^-,i}}\right)\right)$$
$$+\frac{A_g g_g}{FV_i}\left((\phi_i - \phi_{i+1}) + \frac{RT}{F}\ln\left(\frac{c_{Cl^-,i+1}}{c_{Cl^-,i}}\right)\right) - \frac{A_g L_{p,g} c_{Cl^-}^*}{V_i}(\Delta P_i - \Delta P_{i+1}). \qquad (S31)$$

The associated cell volume depends on both hydrostatic and osmotic pressure gradients, as before, with:

$$\frac{dV_i}{dt} = -A_g L_{p,g}(\Delta P_i - \Delta P_{i+1})$$
$$-A_i L_{p,m}\left(\Delta P_i - RT\left(c_{Na^+,i} + c_{K^+,i} + c_{Cl^-,i} + X/V_i - \left(c_{Na^+,e} + c_{K^+,e} + c_{Cl^-,e}\right)\right)\right). \qquad (S32)$$

Following the approach from the mechano-osmotic flow analysis, we explore how an applied stress on the surface of one cell (associated with proliferation of surrounding cells) induces swelling of a neighboring cell. As per the single cell MEO analysis, the system of equations Eqns S23, S29-S32 can be solved either transiently or at steady state; both solutions are shown in Supplementary Fig 6. Simulations reveal that the MEO model predicts the same trends as the mechano-osmotic model discussed in the main paper text (Fig 2): the volume of a loaded cell reduces with increasing applied stress and its neighbor increasingly swells due to an ion influx via gap junctions (Supplementary Fig 6a). The mechanisms by which ions are driven into the neighbor differ slightly due to the consideration of electrical potentials. The applied stress generates an increase in the cytosolic hydrostatic pressure of the loaded cell, driving advective flow across GJs in accordance with Eqns S29-S31. As K^+ ions are the dominant species, such a flow increases the membrane potential of the connected cell. Due to the increased positive charge (Supplementary Fig 6b), Cl^- ions are drawn into the neighboring cell from

both the loaded cell and the external fluid (Supplementary Fig 6e) to maintain electroneutrality. Solvent then enters the cell to reduce the osmotic pressure gradient, which is further lowered by the continuous influx of fluid from the advective GJ flow (Supplementary Fig 3g). Further, the loss of ions from the loaded cell impairs its ability to retain water relative to an isolated cell (Supplementary Fig 5), again similar to the mechanisms identified from non-electrolyte flow in Fig 2.

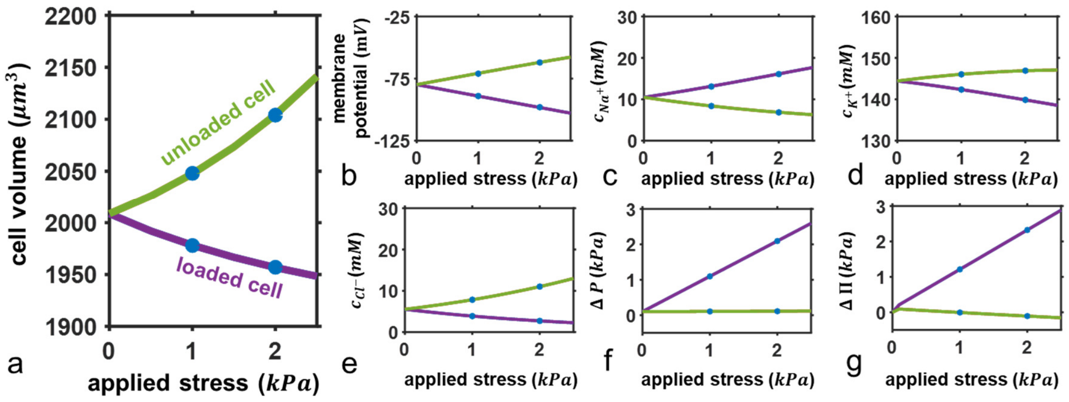

Supplementary Fig 6: ***Role of gap junctions (GJ) in cellular volume control in accordance with MEO model*: In response to applied loading, predictions are shown for a) cell volume, b) membrane potential, c-e) concentrations of Na^+, K^+, and Cl^+, f) hydrostatic pressure and g) osmotic pressure at steady state in a loaded and connected unloaded cell. Markers show corresponding predictions at steady state from transient analysis.**

Supplementary Note 9. Spatial variance in external solute concentrations

In our analyses we implicitly assume that the extracellular ion concentrations are spatially uniform. However, within multi-cellular organoids there may also be local interstitial osmotic perturbations that can influence cell behavior. Further, in large non-vascularized cell clusters there may be an unequal distribution of external solutes. Thus, here we introduce an extension to the MEO model to facilitate exploration of such a non-uniform solute distribution on shrinkage and swelling. Recall that the water flux across the cell membrane is driven by a balance between the internal and external hydrostatic and osmotic pressures, such that $J_{v,m,i} = -L_{p,m}(\Delta P_i - \Delta\Pi_i)$, where $\Delta P_i = P_i - P^{ext}$ and $\Delta\Pi_i = \Pi_i - \Pi^{ext}$. Within the MEO model the osmotic pressure differences are stated explicitly in terms of individual species, with $\Delta\Pi_i = RT(c_{Na^+,i} + c_{K^+,i} + c_{Cl^-,i} + X/V_i - (c_{Na^+,e} + c_{K^+,e} + c_{Cl^-,e}))$. Without loss of generality this difference can be rephrased to consider a local variation in Na^+ concentration $\delta c_{Na^+,e,i}$ such that $\Delta\Pi_i = RT(c_{Na^+,i} + c_{K^+,i} + c_{Cl^-,i} + X/V_i - (c_{Na^+,e} + \delta c_{Na^+,e,i} + c_{K^+,e} + c_{Cl^-,e}))$. As an increase in the interstitial solute concentration will also affect loading on the cell membrane, the associated flux must also be updated to include $\delta c_{Na^+,e,i}$:

$$\frac{dc_{Na^+,i}}{dt} = -\frac{A_i g_{Na}}{FV_i}\left(\phi_i - \frac{RT}{F}\ln\left(\frac{c_{Na^+,e} + \delta c_{Na^+,e,i}}{c_{Na^+,i}}\right)\right) - \frac{3pA_i}{V_i}$$
$$-\frac{A_g g_g}{FV_i}\left((\phi_i - \phi_{i+1}) - \frac{RT}{F}\ln\left(\frac{c_{Na^+,i+1}}{c_{Na^+,i}}\right)\right) - \frac{S_g L_{p,g} c^*_{Na^+}}{V_i}(\Delta P_i - \Delta P_{i+1}). \qquad (S33)$$

Similar modifications could be made to the expressions for K^+ or Cl^-. As shown in Supplementary Fig 7a, the introduction of a small osmotic shock ($5mM$) has a marked, albeit low, influence on the volume of the shocked cell. The sudden change in the external Na^+ concentration causes the osmotic pressure difference to sharply decrease, by $\delta\Delta\Pi_i = RT(5mM) \approx 13\ kPa$ (Supplementary

Fig 7b). However, this perturbation is rapidly balanced by solvent efflux from the cell and solute influx. Overall, it is interesting to note that hydrostatic pressure change that corresponds to such an osmotic pressure fluctuation is quite low (Supplementary Fig 7c), predicted to be on the order of 20 Pa. Naturally, this magnitude depends on effective cell stiffness and resistance to volume change. The steady state volume of the connected neighboring cell is unaltered as the diffusive potential (introduced by the slight variance in the shocked cell's solute concentration) is not sufficient to overcome the balance of electrical potentials. The same behavior may be explored with the mechano-osmotic model presented in the main paper text by similarly perturbing the external osmotic pressure Π^{ext} in Eqns 4 and 6.

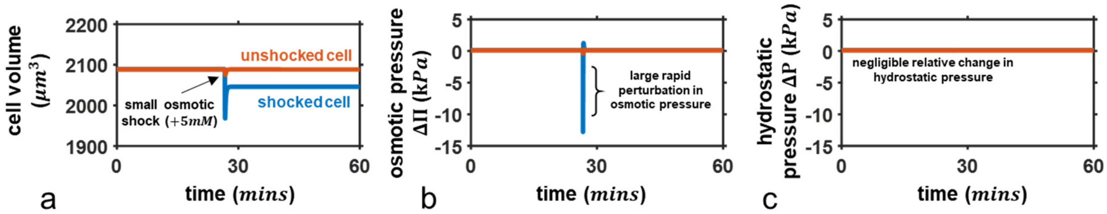

Supplementary Fig 7: ***Response of connected cells to a sudden local osmotic shock*****: Influence on a) cell volume, b) the hydrostatic pressure difference ΔP and c) the osmotic pressure difference $\Delta\Pi$.**

Supplementary Note 10. Spatial variance in external hydrostatic pressure

Beyond osmotic pressure gradients, our analysis also implicitly assumes that the extracellular hydrostatic fluid pressure P^{ext} is spatially uniform (i.e. $P_i^{ext} = P_{i+1}^{ext} = P^{ext}$) and that cell loading is predominantly attributed to solid stress associated with cluster growth. However, within proliferating tumors there may also be local interstitial pressure gradients that can influence cell behavior. Thus, here we extend our formulations to facilitate exploration of their influence on cell shrinkage and swelling. As highlighted in the previous section, the water flux across the cell membrane $J_{v,m,i} = -L_{p,m}(\Delta P_i - \Delta\Pi_i)$, where $\Delta P_i = P_i - P^{ext}$. Without loss of generality this flux can be rephrased such that $J_{v,m,i} = -L_{p,m}\left(\Delta P_i - \delta P_i^{ext} - \Delta\Pi\right)$, where δP_i^{ext} is a hydrostatic pressure perturbation acting only on cell i. Thus, extending Eqn S32, the change in cell volume may be expressed as:

$$\frac{dV_{c,i}}{dt} = -A_g\omega_g(\Delta P_i - \Delta P_{i+1}) \\ -A_i\omega_c(\Delta P_i - \delta P^{ext} - \Delta\Pi_i). \qquad (S34)$$

As an increase in the interstitial fluid pressure will also affect loading on the cell membrane, the mechanical force balance must also be updated, whereby

$$\sigma_i = \frac{K}{2}\left(\frac{r_{c,i}^2}{r_0^2} - 1\right) + \sigma_{a,i} \\ = \frac{(\Delta \mathrm{P}_i - \delta P^{ext} - \sigma_{g,i})r_{c,i}}{2h_i}. \qquad (S35)$$

The introduction of a local hydrostatic pressure perturbation ($\delta P^{ext} = 1\,kPa$) is shown in Supplementary Fig 8. In the absence of gap junctions, our model predicts that (unlike solid stress) an additional fluid pressure does not cause a change in cell volume (Supplementary Fig 8d). Although the increase in external hydrostatic pressure should drive water into the cell, swelling is opposed by the compressive load that the same interstitial pressure imposes on the cell membrane. However, the internal hydrostatic pressure of the loaded cell increases (by a magnitude equal to δP^{ext}). When GJs become permeable, because the hydrostatic pressure of the loaded cell is higher than that of its neighbor, advective flow drives solutes into the neighboring cell, lowering its membrane potential (Supplementary Fig 8b). The loaded cell then shrinks due to water loss, though the electroneutrality condition and electrical potentials limit the volume reduction

(Supplementary Fig 8a). This mechanism is similar to that outlined for solid stress loading in Section S8, whereby the increasing ion concentration in the neighboring cell causes it to swell. Of note, the magnitude of the volume differences from local hydrostatic (δP_i^{ext}) and solid stress ($\sigma_{g,i}$) loading are equivalent (Supplementary Fig 8c). Further, the combined analysis in this and the previous section highlight that an equivalent osmotic and hydrostatic pressure load do not have an identical influence on cellular volume. In terms of volume reduction in the locally loaded cell, an osmotic pressure induced by a $5mM$ solute perturbation ($\sim 10\,kPa$) is found to be broadly equivalent to a hydrostatic pressure 10-fold lower ($\delta P_i^{ext} = 1\,kPa$). However, the impact on neighboring connected cells is markedly different. The same behavior may be explored with the mechano-osmotic model presented in the main paper text by similarly perturbing the external hydrostatic pressure by δP^{ext} in Eqns 3 and 4. Similarly, the extension can also be incorporated to the continuum-level model via inclusion of δP^{ext} in the membrane-specific term within Eqn 9. In this instance the hydrostatic perturbation $\delta P^{ext}(r)$ would need to be spatially defined in a similar context to $\sigma_g(r)$.

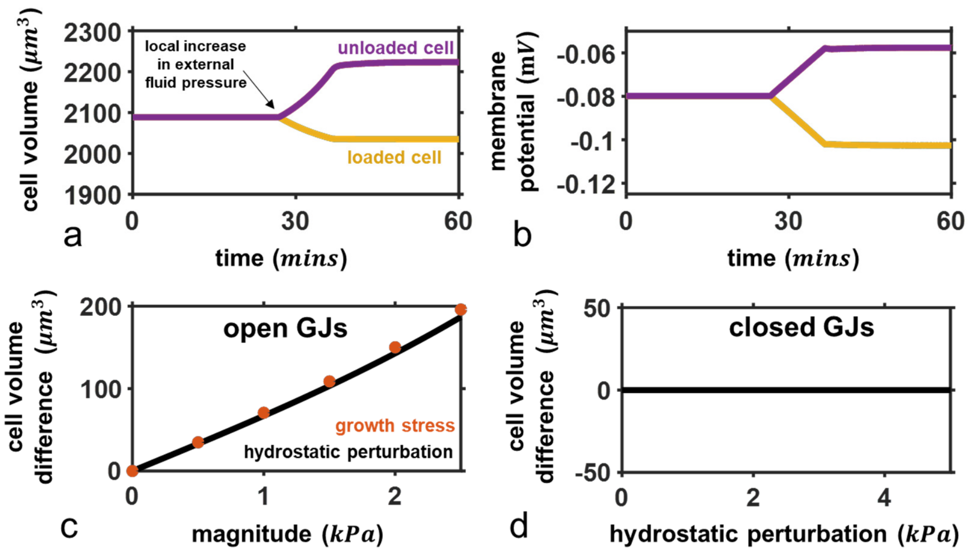

Supplementary Fig 8: ***Response of connected cells to a local differences in hydrostatic pressure*****: Influence on a) cell volume and b) membrane potential; c) Predicted difference in cell volumes ($\Delta V = V_2 - V_1$) for local hydrostatic (δP_i^{ext}) and solid stress ($\sigma_{g,i}$) loading with open GJs; d) Influence of a hydrostatic pressure perturbation on cell volume when GJs are closed.**

Supplementary Note 11. Validation of experimental nuclear measurements

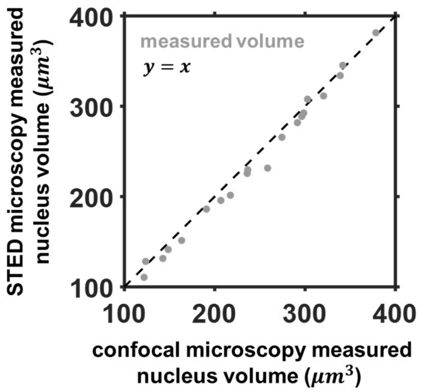

Supplementary Fig 9. ***Validation of nuclear measurements*****. Comparison of the volume measurements using stimulated emission depletion (STED) microscopy with a super-resolution mode with isotropic resolution in x, y and z, and laser scanning confocal microscopy. Nuclear volume of single MCF10A cells is measured by STED and confocal microscopy, showing consistency between two methods.**

Supplementary Note 12. Determination of local solid growth stress in proliferating organoid

In our experimental system single cells were seeded in Matrigel/alginate hydrogels, which had a shear modulus of approximately 300 Pa. The isolated cells proliferated to achieve a small cluster by day 3 (Supplementary Fig 10a) and continued to grow into a larger cluster on day 5. To characterize the stresses introduced by growth, we develop a finite element of model of the proliferative cluster and simulate its deformation of the surrounding hydrogel. We adopt the multiplicative decomposition of the deformation gradient $\mathbf{F}$ into an elastic tensor $\mathbf{F}_e$ and a growth tensor $\mathbf{F}_g$ as proposed by Rodriguez *et al.* (1994) [38], such that $\mathbf{F} = \mathbf{F}_e\mathbf{F}_g$. The growth tensor can be expressed by $\mathbf{F}_g = \lambda_g\mathbf{I}$, where λ_g is the growth stretch and $\mathbf{I}$ is the second order identity tensor. Cluster growth from a single cell to a spheroid with a diameter of approximately 66.8 μm (Supplementary Fig 10a) identifies that $\lambda_g \approx 4.4$, assumed to increase linearly from days 0-5. With this definition of the growth tensor, we can then determine the elastic component of the deformation gradient via $\mathbf{F}_e = \mathbf{F}\,\mathbf{F}_g^{-1}$. The mechanical behavior of the hydrogel and spheroid may then be described by a Neo-Hookean hyperelastic formulation, with a Cauchy stress given by:

$$\boldsymbol{\sigma} = \frac{G}{J_e}\left(\overline{\mathbf{B}}_e - \frac{1}{3}tr(\overline{\mathbf{C}}_e)\mathbf{I}\right) + \kappa(J_e - 1)\mathbf{I}, \qquad (S36)$$

where J_e is the determinant of the elastic component of the deformation gradient, $\overline{\mathbf{B}}_e = J_e^{-\frac{2}{3}}\mathbf{F}_e\mathbf{F}_e^{\mathrm{T}}$ and $\overline{\mathbf{C}}_e = J_e^{-\frac{2}{3}}\mathbf{F}_e^{\mathrm{T}}\mathbf{F}_e$ are the left and right Cauchy-Green tensors, respectively, G is the material shear modulus, and κ is the material bulk modulus. Our hydrogels have a shear modulus $G_h = 300\ Pa$ and we assume a bulk modulus of $\kappa_h = 500\ Pa$ in line with previously reported values[39]. We assume an effective cell shear and bulk modulus of $G_h = 385\ kPa$ and $\kappa_h = 833\ Pa$, in accordance with our previous work[21]. Simulations suggest that with increasing cluster growth, the surrounding hydrogel becomes increasingly stretched (Supplementary Fig 10b), such that on day 5 a pressure $P \approx 550\ Pa$ is applied (Supplementary Fig 10c). Recently, Dolega *et al.* (2017)[40] demonstrated that the pressure in a proliferative cell cluster under applied loading is spatially non-uniform by experimentally measuring the deformation of polyacrylamide beads embedded within the spheroid; the core stress was identified to be approximately equal to the applied load with a 2- to 3-fold reduction at the periphery, approximately following a linear distribution at intermediate locations. Our FE model predictions therefore suggest that the pressure acting on (uniformly) on the surface of individual cells in our day-5 system linearly varies from $\sigma_g^{max} = 550\ Pa$ at the spheroid core to $\sigma_g^{min} = 200\ Pa$ at the periphery (Supplementary Fig 10d). This solid compressive stress enters the continuum framework via an expansion of Eqn 3 with $\sigma(r) = (\Delta P(r) - \sigma_g(r))/2h_i$. Following the continuum analysis from the main manuscript (Fig 3), we find that our model provides excellent agreement with our experimentally observed nuclear volumes at days 3 and 5 of growth (Supplementary Fig 10e-f). The experimentally measured volumes were observed to be spatially uniform on day 3, indicating the applied solid stress is also uniform and can be motivated directly from growth predictions (Supplementary Fig 10c).

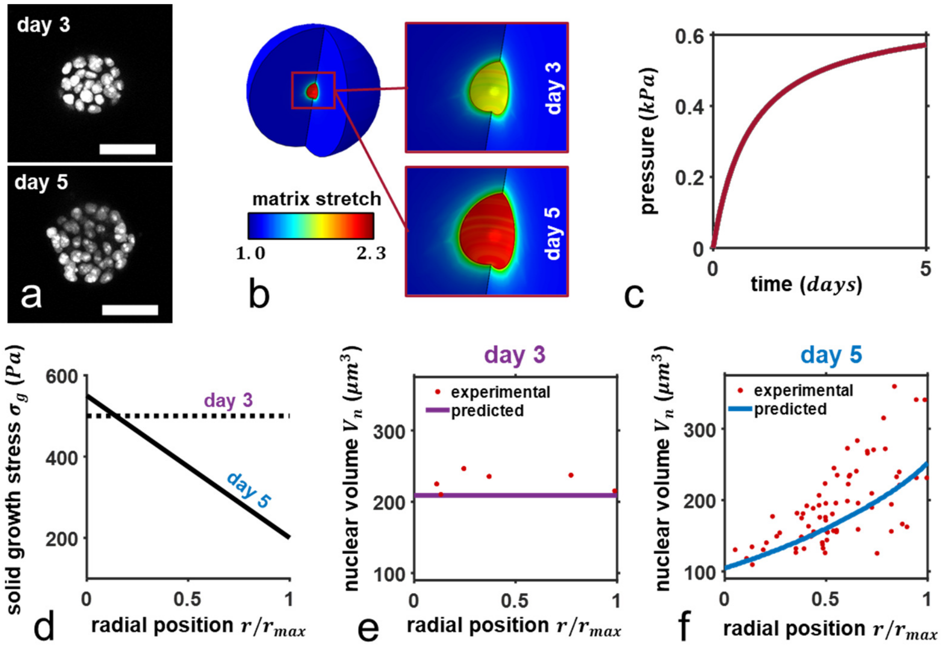

Supplementary Fig 10: ***Prediction of spheroid surface pressure due to growth:*** **a) Cross-section images of GFP-NLS-labelled MCF10A cells at day 3 and 5. Scale bar 50 μm; b) Predicted matrix deformation from growth simulations using finite element analysis. Contours show max principal stretch in the hydrogel; c) Predicted evolution of spheroid surface pressure imposed by hydrogel during growth; d) Applied solid growth stress σ_g (r) is highest at the core and spatially non-uniform; Predicted and experimental spatial cell and nuclear volumes under control conditions on e) day 3 and f) day 5 ($n > 3$).**

Supplementary Note 13. Cell volume varies spatially in breast cancer biopsy sample

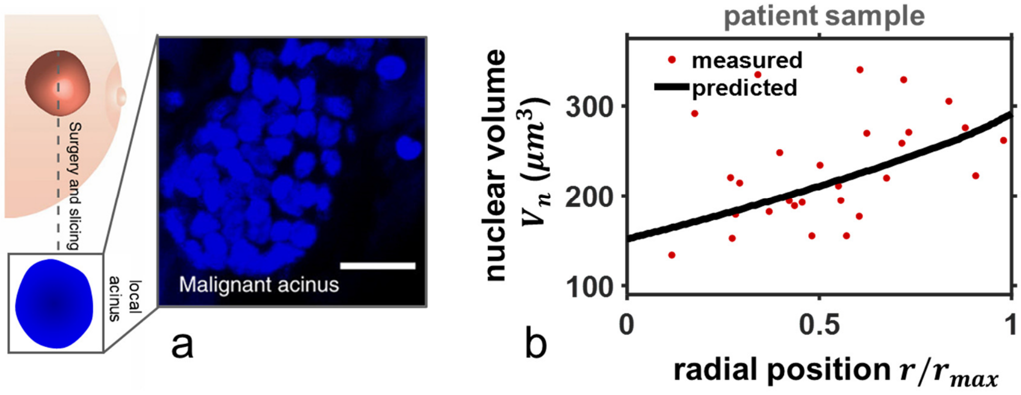

Supplementary Fig 11: ***Characterization of cell volume heterogeneity in patient samples*****. a) Schematic of a tumor biopsy from a breast cancer patient with fluorescent image showing cell in a local malignant acinus. Adapted from our previous work[41]. Scale bar=50 μm; b) Predicted and measured ($n = 3$) spatial cell and nuclear volumes from malignant acinus. For these simulations the reference cell volume $r_0 = 7.5\ \mu m$ and the solid growth stress at the core $\sigma_g(0) = 450\ Pa$. All other parameters remain as reported for the experimental 3D model.**

Supplementary Note 14. Collagen/Matrigel system prior to stress-release

To explore the influence of intra-spheroid stress (σ_g) we performed additional experiments whereby organoids were cultured in a collagen (3.5 $mg\ ml^{-1}$) / Matrigel (0.5 $mg\ ml^{-1}$) matrix (C/M). Cell locations were classified by position within the spheroid: inner ($r/r_{max} \leq 0.25$), inner-mid ($0.25 < r/r_{max} \leq 0.5$), outer-mid ($0.5 < r/r_{max} \leq 0.75$), and outer ($r/r_{max} \geq 0.75$). Comparison with observed day-5 nucleus volumes (Supplementary Fig 12a) from the Matrigel/alginate (M/A) system revealed the volume differences were not significant ($P > 0.05$). Further, our model predictions were also found to lie within the interquartile range (between the first and third quartiles) of both systems (Supplementary Fig 12b), indicating no parameter adjustment is required to capture nuclear volumes across these M/A and C/M systems.

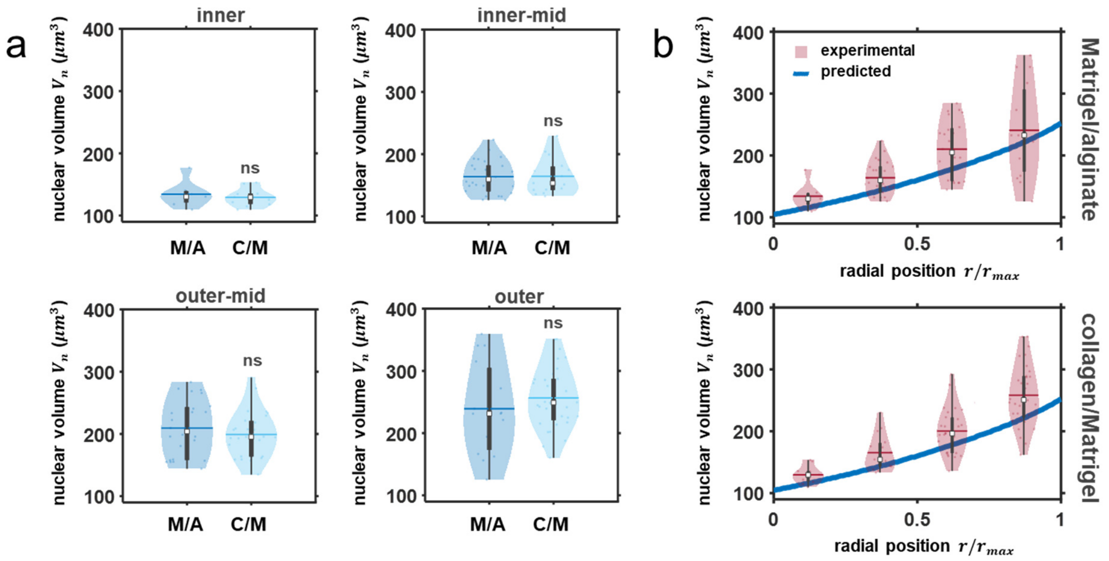

Supplementary Fig 12: ***Nuclear volumes in different systems.*** **a) Comparison of nuclear volume of cells at the inner, inner-mid, outer-mid, and outer organoid regions at day 5 of growth in the Matrigel/alginate (M/A) and collagen/Matrigel (C/M) systems ($n = 6$ multi-cellular clusters over 3 independent experiments). The boxes represent the interquartile range between the first and third quartiles, whereas the whiskers represent the 95% and 5% values, the squares represent the median, the horizontal lines show the mean, and the shaded region bounds indicate the maxima and minima. A two-tailed Student's t-test was used when comparing the difference between two groups, ns: $P > 0.05$; b) Predicted and experimental (day 5) spatial nuclear volumes in the M/A and C/M systems. Experimental distributions plotted at mid-point of associated range.**

Supplementary Note 15. Analytical solutions at limits of gap junction permeability

From our continuum formulation, it is possible to analytically derive solutions at the limits of gap junction permeability. Considering again the expression for the number of cellular ions at position r:

$$\frac{\partial N(r)}{\partial t} = A_g \frac{r_0^2}{r^2}\left[\frac{\Pi^{ext}}{RT} L_{p,g} \frac{\partial}{\partial r}\left(r^2 \frac{\partial \Delta P(r)}{\partial r}\right) + \omega_g \frac{\partial}{\partial r}\left(r^2 \frac{\partial \Delta\Pi(r)}{\partial r}\right)\right]$$
$$-4\pi r_c^2(r)\Big(\big(\omega_{ms}(\sigma(r)) + \omega_l + \gamma\big)\Delta\Pi(r) - \gamma\Delta\Pi_c\Big), \qquad (S37)$$

and cell volume is described by

$$\frac{\partial V_c(r)}{\partial t} = A_g L_{p,g} \frac{r_0^2}{r^2}\frac{\partial}{\partial r}\left(r^2 \frac{\partial\big(\Delta P(r)\big)}{\partial r}\right)$$
$$-4\pi r_c^2(r) L_{p,m}\big(\Delta P(r) - \Delta\Pi(r)\big),. \qquad (S38)$$

At steady state, $\frac{\partial N(r)}{\partial t} = \frac{\partial V_c(r)}{\partial t} = 0$, thus considering the limit of zero gap junction permeability (i.e. $\omega_g = L_{p,g} = 0$), Eqn S37 yields:

$$\Delta\Pi(\mathrm{r}) = \frac{\gamma\Delta\Pi_c}{\omega_{ms}(\sigma(r)) + \omega_l + \gamma}. \qquad (S39)$$

Under these conditions, at steady state $\Delta\Pi(\mathrm{r}) = \Delta P(r)$ (via Eqn S38), then mechanical equilibrium dictates:

$$\sigma(\mathrm{r}) = \frac{K}{2}\left(\frac{r_c^2(r)}{r_0^2} - 1\right) + \sigma_a$$
$$= \frac{\big(\Delta\Pi(r) - \sigma_g(r)\big) r_c(r)}{2h}. \qquad (S40)$$

Therefore, spatial cell volume can obtained for a given solid stress distribution $\sigma_g(r)$.

Next, considering the limit of infinite gap junction permeability ($\omega_g = L_{p,g} = \infty$) mandates that $\frac{\partial}{\partial r}\left(r^2 \frac{\partial \Delta\Pi(r)}{\partial r}\right) = \frac{\partial}{\partial r}\left(r^2 \frac{\partial \Delta \mathrm{P}(r)}{\partial r}\right) = 0$. As such, $\Delta\Pi(r)$ must be constant and spatially uniform (i.e. $\Delta\Pi(\mathrm{r}) = \Delta\Pi^*$). We can then multiply Eqn S37 by r^2 and integrate between 0 and r_{max} to obtain:

$$4\pi \int_0^{r_{max}} r^2\, r_c^2(r)\big(\omega_{ms}(\sigma(r)) + \omega_l + \gamma\big)\Delta\Pi^* dr$$
$$= 4\pi(\Delta\Pi_c\gamma)\int_0^{r_{max}} r^2\, r_c^2(r) dr, \qquad (S41)$$

which leads to:

$$\Delta\Pi^* = \frac{\gamma\Delta\Pi_c}{\gamma + \omega_l + \frac{<\omega_{ms}\big(\sigma(r)\big)\, r_c^2(r)>}{<r_c^2(r)>}}, \qquad (S42)$$

where $< r_c^2(r) > = \int_0^{r_{max}} r^2\, r_c^2(r) dr$

and $< f\big(\sigma(r)\big)\, r_c^2(r) > = \int_0^{r_{max}} f\big(\sigma(r)\big)\, r^2\, r_c^2(r) dr$.

Supplementary References

1. Kedem, O. & Katchalsky, A. Thermodynamic analysis of the permeability of biological membranes to non-electrolytes. *BBA - Biochim. Biophys. Acta* **27**, 229–246 (1958).
2. Onsager, L. Reciprocal relations in irreversible processes. I. *Phys. Rev.* **37**, 405–426 (1931).
3. Staverman, A. J. Non-equilibrium thermodynamics of membrane. *Trans. Faraday Soc.* **48**, 176–185 (1952).
4. Smyth, J. W. & Shaw, R. M. The gap junction life cycle. *Hear. Rhythm* **9**, 151–153 (2012).
5. Harris, A. L. Emerging issues of connexin channels : biophysics fills the gap. *Q. Rev. Biophys.* **34**, 325–472 (2002).
6. Gao, J. *et al.* Lens intracellular hydrostatic pressure is generated by the circulation of sodium and modulated by gap junction coupling. *J. Gen. Physiol.* **137**, 507–520 (2011).
7. Agre, P. *et al.* Aquaporin water channels - From atomic structure to clinical medicine. *Journal of Physiology* **542**, 3–16 (2002).
8. Kung, C. A possible unifying principle for mechanosensation. *Nature* **436**, 647–654 (2005).
9. Sukharev, S. I., Sigurdson, W. J., Kung, C. & Sachs, F. Energetic and spatial parameters for gating of the bacterial large conductance mechanosensitive channel, MscL. *J. Gen. Physiol.* **113**, 525–539 (1999).
10. Jiang, H. & Sun, S. X. Cellular pressure and volume regulation and implications for cell mechanics. *Biophys. J.* **105**, 609–619 (2013).
11. Alberts, B. *et al.* Ion Channels and the Electrical Properties of Membranes. in *Molecular Biology of the Cell* (Garland Science, 2002).
12. Glitsch, H. G. & Tappe, A. Change of Na+ pump current reversal potential in sheep cardiac Purkinje cells with varying free energy of ATP hydrolysis. *J. Physiol.* **484**, 605–616 (1995).
13. Cartagena-Rivera, A. X., Logue, J. S., Waterman, C. M. & Chadwick, R. S. Actomyosin Cortical Mechanical Properties in Nonadherent Cells Determined by Atomic Force Microscopy. *Biophys. J.* **110**, 2528–2539 (2016).
14. Kapus, A. & Janmey, P. Plasma Membrane-Cortical Cytoskeleton Interactions: A Cell Biology Approach with Biophysical Considerations. in *Comprehensive Physiology* **3**, 1231–1281 (John Wiley & Sons, Inc., 2013).
15. Shi, Z. & Baumgart, T. Membrane tension and peripheral protein density mediate membrane shape transitions. *Nat. Commun.* **6**, 1–8 (2015).
16. Smeets, B., Cuvelier, M., Pešek, J. & Ramon, H. The Effect of Cortical Elasticity and Active Tension on Cell Adhesion Mechanics. *Biophys. J.* **116**, 930–937 (2019).
17. Tinevez, J. Y. *et al.* Role of cortical tension in bleb growth. *Proc. Natl. Acad. Sci. U. S. A.* **106**, 18581–18586 (2009).
18. Evans, E. & Yeung, A. Apparent viscosity and cortical tension of blood granulocytes determined by micropipet aspiration. *Biophys. J.* **56**, 151–160 (1989).
19. Koay, E. J., Shieh, A. C. & Athanasiou, K. A. Creep indentation of single cells. *J. Biomech. Eng.* **125**, 334–341 (2003).
20. Stewart, M. P. *et al.* Hydrostatic pressure and the actomyosin cortex drive mitotic cell rounding. (2011). doi:10.1038/nature09642
21. Shenoy, V. B., Wang, H. & Wang, X. A chemo-mechanical free-energy-based approach to model durotaxis and extracellular stiffness-dependent contraction and polarization of cells. *Interface Focus* **6**, 20150067 (2016).

22. McEvoy, E., Shishvan, S. S., Deshpande, V. S. & McGarry, J. P. Thermodynamic Modeling of the Statistics of Cell Spreading on Ligand-Coated Elastic Substrates. *Biophys. J.* **115**, 2451–2460 (2018).

23. Shraiman, B. I. Mechanical feedback as a possible regulator of tissue growth. *Proc. Natl. Acad. Sci. U. S. A.* **102**, 3318–3323 (2005).

24. Kalli, M. & Stylianopoulos, T. Defining the role of solid stress and matrix stiffness in cancer cell proliferation and metastasis. *Frontiers in Oncology* **8**, (2018).

25. Mathias, R. T., White, T. W. & Brink, P. R. Chapter 3 The Role of Gap Junction Channels in the Ciliary Body Secretory Epithelium. *Current Topics in Membranes* **62**, 71–96 (2008).

26. Nielsen, M. S. *et al.* Gap junctions. *Compr. Physiol.* **2**, 1981–2035 (2012).

27. Stavarache, I. *et al.* Electrical behavior of multi-walled carbon nanotube network embedded in amorphous silicon nitride. *Nanoscale Res. Lett.* **6**, 87 (2011).

28. Marrink, S.-J. & Berendsen, H. J. C. *Simulation of Water Transport through a Lipid Membrane*. *J. Phys. Chem* **98**, (1994).

29. Kumar, R., Saha, S. & Sinha, B. Cell spread area and traction forces determine myosin-II-based cortex thickness regulation. *Biochim. Biophys. Acta - Mol. Cell Res.* **1866**, 118516 (2019).

30. Kuznetsova, T. G., Starodubtseva, M. N., Yegorenkov, N. I., Chizhik, S. A. & Zhdanov, R. I. Atomic force microscopy probing of cell elasticity. *Micron* **38**, 824–833 (2007).

31. Alberts, B. *et al. Molecular biology of the cell*. (Garland Science, 2002).

32. Grosell, M. Intestinal anion exchange in marine fish osmoregulation. *Journal of Experimental Biology* **209**, 2813–2827 (2006).

33. Larsen, E. H., Møbjerg, N. & Nielsen, R. Application of the Na+ recirculation theory to ion coupled water transport in low- and high resistance osmoregulatory epithelia. *Comparative Biochemistry and Physiology - A Molecular and Integrative Physiology* **148**, 101–116 (2007).

34. Keener, J. P. & Sneyd, J. *Mathematical physiology*. (Springer, 1998).

35. Mori, Y. Mathematical properties of pump-leak models of cell volume control and electrolyte balance. *J. Math. Biol.* **65**, 875–918 (2012).

36. Patel, A., Demolombe, S. & Honoré, E. Piezo1 Ion Channels: An alternative to force. *Elife* **4**, (2015).

37. Kay, A. R. How Cells Can Control Their Size by Pumping Ions. *Front. Cell Dev. Biol.* **5**, 41 (2017).

38. Rodriguez, E. K., Hoger, A. & McCulloch, A. D. Stress-dependent finite growth in soft elastic tissues. *J. Biomech.* **27**, 455–467 (1994).

39. Steinwachs, J. *et al.* Three-dimensional force microscopy of cells in biopolymer networks. *Nat. Methods* **13**, 171–176 (2016).

40. Dolega, M. E. *et al.* Cell-like pressure sensors reveal increase of mechanical stress towards the core of multicellular spheroids under compression. *Nat. Commun.* **8**, (2017).

41. Han, Y. L. *et al.* Cell swelling, softening and invasion in a three-dimensional breast cancer model. *Nat. Phys.* (2019). doi:10.1038/s41567-019-0680-8
