# Supplementary material for: Gap junctions amplify spatial variations in cell volume in proliferating tumor spheroids
Source: Nat Commun. 2020 Dec 1;11:6148. doi: 10.1038/s41467-020-19904-5 (PMC7708487; doi:10.1038/s41467-020-19904-5)
Supplement: Supplementary file 3 — Reporting Summary [file 41467_2020_19904_MOESM3_ESM.pdf]

nature research

Corresponding author(s): Vivek B. Shenoy

Last updated by author(s): Oct 12, 2020

Reporting Summary

Nature Research wishes to improve the reproducibility of the work that we publish. This form provides structure for consistency and transparency in reporting. For further information on Nature Research policies, see Authors & Referees and the Editorial Policy Checklist.

Statistics

For all statistical analyses, confirm that the following items are present in the figure legend, table legend, main text, or Methods section.

n/a	Confirmed	
☐	☒	The exact sample size (*n*) for each experimental group/condition, given as a discrete number and unit of measurement
☐	☒	A statement on whether measurements were taken from distinct samples or whether the same sample was measured repeatedly
☐	☒	The statistical test(s) used AND whether they are one- or two-sided<br>*Only common tests should be described solely by name; describe more complex techniques in the Methods section.*
☒	☐	A description of all covariates tested
☒	☐	A description of any assumptions or corrections, such as tests of normality and adjustment for multiple comparisons
☒	☐	A full description of the statistical parameters including central tendency (e.g. means) or other basic estimates (e.g. regression coefficient) AND variation (e.g. standard deviation) or associated estimates of uncertainty (e.g. confidence intervals)
☒	☐	For null hypothesis testing, the test statistic (e.g. *F*, *t*, *r*) with confidence intervals, effect sizes, degrees of freedom and *P* value noted<br>*Give P values as exact values whenever suitable.*
☒	☐	For Bayesian analysis, information on the choice of priors and Markov chain Monte Carlo settings
☐	☒	For hierarchical and complex designs, identification of the appropriate level for tests and full reporting of outcomes
☒	☐	Estimates of effect sizes (e.g. Cohen's *d*, Pearson's *r*), indicating how they were calculated

Our web collection on statistics for biologists contains articles on many of the points above.

Software and code

Policy information about availability of computer code

Data collection	The particle tracking (cells and particles within the cytoplasm) and volume calculation are processed with customized code in Matlab 2017a. Matlab 2019a and COMSOL 5.4 are used for simulating cell behavior, and code is openly available on Github (https://github.com/EoinMcEvoy).
Data analysis	OriginLab 8.0 and Matlab 2017a are used to analyze the experimental data in this study.

For manuscripts utilizing custom algorithms or software that are central to the research but not yet described in published literature, software must be made available to editors/reviewers. We strongly encourage code deposition in a community repository (e.g. GitHub). See the Nature Research guidelines for submitting code & software for further information.

Data

Policy information about availability of data

All manuscripts must include a data availability statement. This statement should provide the following information, where applicable:

- Accession codes, unique identifiers, or web links for publicly available datasets
- A list of figures that have associated raw data
- A description of any restrictions on data availability

The datasets generated during and/or analysed during the current study are available from the corresponding author on reasonable request.

Field-specific reporting

Please select the one below that is the best fit for your research. If you are not sure, read the appropriate sections before making your selection.

☒ Life sciences ☐ Behavioural & social sciences ☐ Ecological, evolutionary & environmental sciences

For a reference copy of the document with all sections, see nature.com/documents/nr-reporting-summary-flat.pdf

Life sciences study design

All studies must disclose on these points even when the disclosure is negative.

Sample size	Five to fifteen microtumors are measured in each experiment. The precise number used are given in the figure legend.
Data exclusions	For imaging process, only clusters started from a single cell were examined to synchronize the growth of the clusters. To avoid the effects from other clusters, clusters that have close neighbor clusters were also excluded.
Replication	All the measurements were done at least in three independent experiments to verify the reproducibility of the experimental findings (precise number are given in the figure legend), and the results were consistent.
Randomization	Analyzed microtumors are randomly chosen.
Blinding	The investigators were blinded to group allocation during data collection and analysis for osmotic pressure and drug perturbation results.

Reporting for specific materials, systems and methods

We require information from authors about some types of materials, experimental systems and methods used in many studies. Here, indicate whether each material, system or method listed is relevant to your study. If you are not sure if a list item applies to your research, read the appropriate section before selecting a response.

Materials & experimental systems

n/a	Involved in the study	
☒	☐	Antibodies
☐	☒	Eukaryotic cell lines
☒	☐	Palaeontology
☒	☐	Animals and other organisms
☒	☐	Human research participants
☒	☐	Clinical data

Methods

n/a	Involved in the study	
☒	☐	ChIP-seq
☒	☐	Flow cytometry
☒	☐	MRI-based neuroimaging

Eukaryotic cell lines

Policy information about cell lines

Cell line source(s)	Cell lines were purchased from ATCC
Authentication	None of the cell lines used were authenticated because they are freshly purchased.
Mycoplasma contamination	Cell lines were not tested for mycoplasma contamination because they are freshly purchased.
Commonly misidentified lines (See ICLAC register)	No commonly misidentified lines were used in this research.
